# Whether Intracranial Aneurysm Could Be Well Treated by Flow Diversion: A Comprehensive Meta-Analysis of Large-Sample Studies including Anterior and Posterior Circulation

**DOI:** 10.1155/2021/6637780

**Published:** 2021-03-08

**Authors:** Yingjin Wang, Changwei Yuan, Shengli Shen, Liqing Xu, Hongzhou Duan

**Affiliations:** Department of Neurosurgery, Peking University First Hospital, No. 8 Xishiku Street, Xicheng District, Beijing 100034, China

## Abstract

**Background:**

Flow diversion (FD) has become a widely adopted treatment method for intracranial aneurysms in the clinic, but a comprehensive meta-analysis of large-sample studies including anterior and posterior circulation is still lacking.

**Methods:**

The PubMed, Embase, Web of Science, and Cochrane databases were searched between January 1, 2008, and December 1, 2019. A random-effect model was used to calculate the efficacy and safety data as well as 95% confidence intervals (CIs).

**Results:**

The pooled sample size of all included studies was 6695 patients; the mean age was 55.5 years old, with a total of 7406 aneurysms. For efficacy, the complete occlusion rate in angiographic follow-up (AFU) at 6 months was 78% (95% CI, 0.77, 0.80), and the AFU rate at 6-12 months was 90% (95% CI, 0.88, 0.92). For safety, the hemorrhagic event rate was 2%, the ischemic event rate was 5%, and the mortality rate was 3%.

**Conclusion:**

FD is an effective and safe treatment for intracranial aneurysm with high complete occlusion rate and acceptable complication rate.

## 1. Background

Over the past three decades, endovascular embolization with or without device assistance has been a widely adopted treatment method for intracranial aneurysms. However, a subgroup of lesions, including fusiform, wide-necked, and large-to-giant aneurysms, continues to present major challenges [1].

Fortunately, flow diversion (FD) is designed to provide sufficient metal coverage across the neck of the aneurysm to physiologically exclude the lesion from the circulation [2]. More importantly, flow diverters induce thrombosis into the aneurysmal sac while preserving physiological flow in the parent vessel and adjacent branches [3]. This excellent function is based on the special structure of a braided mesh cylinder composed of individual platinum and cobalt chromium microfilaments [4]. From the first case of a pipeline implantation [2] to the present, a large number of studies have reported the efficacy of flow diversion, and more advanced FD techniques have been designed and applied in clinical treatment, including silk FD and a flow-redirection endoluminal device [5, 6]. Although a series of reviews and meta-analyses have been published over the past years [7–11], a comprehensive meta-analysis with a large sample data is still lacking.

The objective of this meta-analysis is to calculate a relatively more reliable result of the efficacy and safety of FD based on a large sample size and detailed data demonstration.

## 2. Methods

### 2.1. Search Strategy

We systematically searched the PubMed, Embase, Web of Science, and Cochrane databases between January 1, 2008 (the publication date of the first pipeline), and December 1, 2019, to identify all relevant articles on flow diversion in intracranial aneurysms. The following keywords were used in our searches: “Flow diversion” OR “*Flow Diverter*” OR “*Flow-Diverting*” OR “*Pipeline*” OR “*PED*” OR “Surpass” OR “*Silk*” OR “*FRED*”; “*Intracranial Aneurysms*” OR “*Artery Aneurysms*” OR “*Cerebral Aneurysms*” OR “*Communicating Artery*”, with no language restrictions. Additionally, we searched the reference lists of the retrieved articles to further identify possibly eligible studies. The searches were performed independently by two investigators (Y.J.W. and C.W.Y), and any discrepancies were solved via discussion until a consensus was reached.

### 2.2. Selection Criteria and Quality Evaluation

Studies were included in the meta-analysis if they fulfilled the following criteria: (1) flow diversions were used for the treatment of intracranial aneurysms; (2) the outcome data included occlusion rates, complication rates, and follow-up time; and (3) sample size > 100. Furthermore, studies were excluded if (1) they were case reports or case series; (2) they used a therapy method combined with FD or other endovascular materials, such as coils; (3) the studies did not report the occlusion rate in the article, sometimes the studies were retrospectively designed as a comparison between occluded and nonoccluded groups; and (4) the last angiographic follow-up was short term (several weeks or <1 month).

The quality evaluation for each study was performed using the guidelines from the Strengthening of Reporting of Observational studies in Epidemiology (STROBE) Statement, including a 22-item checklist (see Supplement 1). This study was planned and executed in accordance with the Preferred Reporting Items for Systematic Reviews and Meta-Analyses (PRISMA) guidelines [12].

### 2.3. Data Extraction

All data were independently extracted by two authors (Y.J.W. and C.W.Y.) using a customized data collection form. When necessary, the original authors were contacted for supplementary information. The following data were extracted: first author's name, publication year, country, patient number, aneurysm number, FD types, mean age, gender, design, clinical presentations, and aneurysm characteristics (size, location, and morphology). The outcome data included complete occlusion number/rate and complication events/rates.

### 2.4. Statistical Analysis and Outcome Assessment

To coordinate the outcome data from the included studies, we predefined the criterion of occlusion. We defined complete occlusion (CO, 100% occlusion) as a valid therapeutic outcome. In addition, after data extraction, we predefined three groups in terms of postoperative angiographic follow-up (AFU): (1) AFU < 6 months, (2) AFU of 6 months, and (3) AFU between 6 and 12 months. The data from studies with a strict AFU of 6 months were sorted into group (2). In addition, the studies that did not have a preset AFU time period were sorted into group (1) or (3) according to their mean or last AFU time.

In the safety analysis, we examined several common complications: hemorrhagic events, ischemic events, and mortality rates. We defined hemorrhagic events (HEs) as subarachnoid hemorrhage (SAH), cerebral hemorrhage, intraventricular hemorrhage, and defined ischemic events (IEs) including any instances of cerebral embolization/thrombosis and transient ischemic attack (TIA). In terms of mortality, we did not integrate the primary data and merely pooled the records.

In this meta-analysis, a random-effect model was used to calculate the CO rate and all complications rates as well as 95% confidence intervals (CIs). The statistical heterogeneity among the summary data was evaluated using the *I*^2^ statistic [13, 14]. We regarded that *I*^2^ < 40% indicated “heterogeneity might not be important” and *I*^2^ > 75% indicated “considerable heterogeneity” based on the suggestions provided by the *Cochrane Handbook for Systemic Reviews of Interventions* [15]. Heterogeneity was considered statistically significant if *p* < 0.05.

To accurately evaluate the CO rate at the 6-month follow-up, we performed four subgroup analyses based on FD type, age, aneurysm dome size, and single/multicenter design.

## 3. Results

### 3.1. Search and Selection

**Figure 1:**
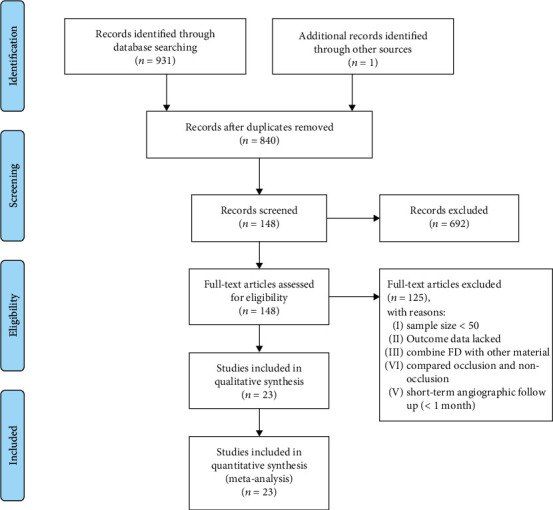
PRISMA flow chart of included eligible studies.

From the primary search of the PubMed, Embase, Web of Science, and Cochrane databases and the manual search of reference lists, 932 potentially eligible records were identified. After screening the titles and abstracts, one hundred forty-eight articles were considered potentially eligible studies. After full-text screening, twenty-three studies [16–38] met the inclusion criteria (Figure 1).

### 3.2. Patient and Aneurysm Characteristics

The pooled sample size of all included studies was 6695 patients with 7406 aneurysms. All the studies had a retrospective design and were published from 2012 to 2019. The mean patient age was 55.5 years old, with a total female rate of 76.8%. The main clinical presentations of the patients were asymptomatic/incidental (42.7%) and headache (25.4%). Ten studies described preoperative modified Rankin Scale (mRS) scores, and thirteen studies listed mRS scores in the postoperative data. The detailed patient data are shown in Table 1.

The characteristic data of the aneurysms are shown in Table 2. All studies were categorized into 4 regions (14 from North America, 5 from Europe, 2 from Asia, and 2 from Latin America). Regarding the sample sources, 17 studies were from multicenter sources, and six were from single-center sources. The mean size of the included aneurysms was 8.3 ± 4.5 mm. Subgroup analysis was performed by dome and neck lengths. The location of the aneurysms was listed by anterior circulation and posterior circulation. The aneurysms from the internal carotid, ophthalmic, cavernous, and clinoid arteries accounted for the main proportion. In terms of morphology, saccular and fusiform were the major sources. The basic characteristic data are shown in Supplement 2.

### 3.3. Efficacy: Complete Occlusion Rate

The postoperative occlusion data in all follow-up periods were extracted in each study. The total complete occlusion rate was pooled from the maximum sample number of the AFU. From the results of the different follow-up periods, the complete occlusion rate for an AFU < 6 months was 68% (95% CI, 0.65, 0.72), the complete occlusion rate for an AFU of 6 months was 78% (95% CI, 0.77, 0.80), and the complete occlusion rate for an AFU of 6-12 months was 90% (95% CI, 0.88, 0.92).

To identify the relationship between the results and some factors (FD type, age, dome size, and data source), a subgroup analysis was performed. The detailed data are shown in Table 3.

### 3.4. Safety: Complication Rate

**Figure 2:**
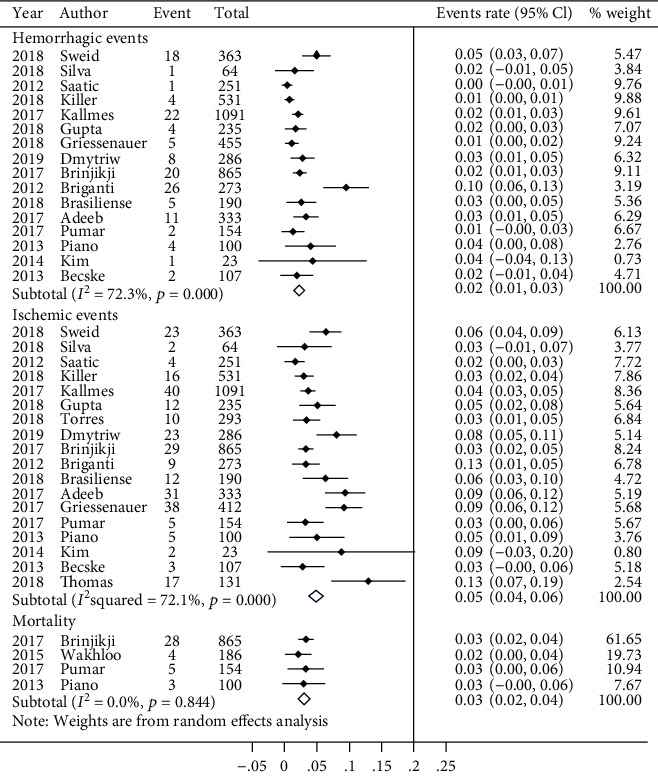
Forest plot of safety data (including hemorrhagic event rate, ischemic event rate, and mortality data).

Data on three postoperative outcomes (HEs, IEs, and mortality) were pooled to evaluate the safety of FD. As shown in Figure 2, the pooled HE rate was 2% (95% CI, 0.02, 0.03), the IE rate was 5% (95% CI, 0.04, 0.06), and the mortality rate was 3% (95% CI, 0.02, 0.04).

### 3.5. Heterogeneity

For occlusion data, the total heterogeneity was negligible (*I*^2^ = 0.0%, *p* = 0.567; *I*^2^ = 9.7%, *p* = 0.341; *I*^2^ = 34.4%, *p* = 0.133, respectively). In terms of the safety outcome, the heterogeneity was moderately high for the HE and IE rates (*I*^2^ = 72.3%, *p* ≤ 0.001; *I*^2^ = 72.1%, *p* ≤ 0.001, respectively). However, for mortality, the heterogeneity was fairly low (*I*^2^ = 0.0%, *p* = 0.844).

## 4. Discussion

This is the first meta-analysis with the largest sample size, in terms of the efficacy and safety of FD, and all the included studies contained at least 100 patients.

### 4.1. Population and Aneurysms

A total of 6695 patients with 7406 aneurysms were included in this meta-analysis. We described the extracted data according to category, including region, clinical presentations, data source design, and aneurysm location/size/morphology. Most patients were identified via medical examinations or headaches. The aneurysms were mainly located in the anterior circulation, in which lesions in the ICA and ophthalmic artery accounted for the main proportion. In terms of morphology, saccular and fusiform structures were more common. Based on a large number of aneurysms, the demonstration of epidemiological features could be more robust and reliable.

To review the published literature in the past decade, Arrese et al. [4] and Brinjikji et al. [8] performed relatively early meta-analyses in 2013, targeting intracranial aneurysms from 897 and 1451 patients, respectively. Subsequently, in the past three years, Cagnazzo et al. [39–42] and Sorenson et al. [43] performed their updated meta-analysis, illustrating detailed outcomes according to aneurysm location from the ACoA, MCA, PCoA, etc. For the recent meta-analysis, they included data with specific locations or morphologies so that their conclusions explained the specific problem and provided detailed evidence, while the sample size of some of their included studies was <10 [40, 44]. Including too many small-sample studies might result in a nonnegligible fluctuation of the results, just as the complete occlusion rate calculated by Kiyofuji et al. [44] was 52% (29-76%), while the record from Cagnazzo et al. [45] was 85.3% (78.2-92.4%). For unruptured nonsaccular intracranial aneurysms of the posterior circulation, the two studies also showed different outcomes. We consider that the pooled results of large sample can weaken the influence of operator's technique, case selection, and operation mode on the results and make the analysis results more objective and reliable.

### 4.2. Occlusion Rate

The evaluation of complete occlusion rate relied on different angiographic follow-up (AFU) periods. It was clear that longer AFU indicated higher occlusion rates. In our study, we strictly pooled the studies with a regular AFU of 6 months, in which the low heterogeneity will guarantee the reliability of the results, and the result showed that the accurate occlusion rate was 78% (75-82%) at the 6-month AFU. Similarly, Brinjikji et al. [8] found a complete occlusion rate (mean AFU of 6 months) of 76% (70-81%), which was consistent with ours. In addition, we separately pooled the data of <6 months' and 6-12 months' group, and the CO rate was 68% (65-72%) and 90% (88-92%), respectively. The higher occlusion rate with time will increase the confidence of clinical use of FD in the future.

The correlation between occlusion rate and dome size was reported in some included studies. It showed that the complete occlusion rate of large (>15 mm) or giant-sized aneurysms (>25 mm) was lower than that of small aneurysms (<7 or <10 mm). The previous reviews [8, 44] also reported similar results to support this trend.

### 4.3. Complications

In this study, we integrated the records with subarachnoid or cerebral or ventricular hemorrhage as hemorrhage events and integrated the records with cerebral embolization/thrombosis, TIA, etc. as ischemic events. Compared with the HE rate, the IE rate was marginally higher (5% vs. 2%). The mortality rate was approximately 3% due to all causes.

Sorenson et al. [43] pooled the periprocedural complication rates of anterior cerebral artery and anterior communicating artery, showing ischemic stroke, hemorrhagic stroke, and mortality with records of 3%, 5%, and 2%, respectively. The result was consistent with ours, while our mortality rate was a little higher. In 2018, Cagnazzo et al. reported that the complication rate of posterior circulation was higher than that of anterior circulation (27% vs. 11.7%). In our meta-analysis, both anterior and posterior circulation aneurysms were included, and the slightly higher mortality might be explained by this. Furthermore, the HE and IE rates were consistent with most of the included studies and some of the published reviews, which confirmed that the application of FD in the treatment of aneurysm was relatively safe.

### 4.4. Quality Evaluation

Nonrandomized and retrospective studies are commonly considered to have low-level evidence, such as the included studies in this meta-analysis. However, most of the included studies had a multicenter design, and the sample size of each study was greater than 100. Therefore, we consider that most of the included studies could meet the medium level of evidence.

### 4.5. Strengths and Limitations

To our knowledge, this study might be the first meta-analysis of flow diversion with the largest sample size, as in each study the sample size was over 100. There are many advantages in our study. Firstly, based on the large sample size, we demonstrate detailed characteristic data of populations and aneurysms. Secondly, we evaluated both the efficacy and safety of FD with detailed and sufficient outcome data. Thirdly, the heterogeneity in the results of occlusion rate was low, which indicates the validity and reliability of the efficacy. Furthermore, PRISMA guidelines were followed to improve the quality of the present analysis and findings reported [12]. Therefore, the pooled occlusion rate in our study is considered to be reliable and accurate, based on the large sample sizes and markedly low heterogeneity.

Regarding the limitations, firstly and most importantly, the analysis model was a single-arm design without a parallel control group. Due to this limitation, this study was a descriptive analysis that merely described the efficacy and safety of FD rather than making a comparison. Secondly, although the detailed characteristics of the patients and aneurysms were listed, no detailed data could be analyzed with more subgroups (such as aneurysm location/morphology and clinical presentations). We excluded <100 sample studies, meanwhile losing more individual data published in case series. While Cagnazzo et al. [40–42, 45] and Sorenson et al. [43] have made up for this deficiency, they included studies with individual data so that the specific subgroup result could be supported. Finally, the heterogeneity in the HE and IE results was still nonnegligible, even though the results were consistent with most multicenter studies.

## 5. Conclusion

Based on the analysis with a large sample size and low heterogeneity, FD might be an effective and safe treatment for intracranial aneurysm with high complete occlusion rate and acceptable complication rate.

## Figures and Tables

**Table 1:** Basic characteristics of patients.

	Mean (SD)	*N* of studies	*N* of patients
Age (year)	55.5 (13.4)	23	6695
Female		23	5031 (75.1%)
Clinical presentation		13	1168
Asymptomatic/incidental		12/13	872 (74.7%)
Headache		11/13	517 (44.3%)
Hemorrhagic lesion		11/13	203 (17.3%)
Ischemic lesion		5/13	54 (4.6%)
Visual change		5/13	78 (6.7%)
Other cranial nerve		6/13	166 (14.2%)
Recurring		4/13	150 (12.8%)
Preoperative mRS		10	2698
mRS 0-2		10/10	2591 (96.0%)
mRS 3-5		10/10	107 (4.0%)
Postoperative mRS		13	2449
mRS 0-2		13/13	2283 (93.2%)
mRS 3-5		13/13	166 (6.8%)

Abbreviations: mRS: modified Rankin Scale.

**Table 2:** Basic characteristics of aneurysms.

Type		*N* of studies	*N* (%) of aneurysms
Region	North America	14	5210 (70.3%)
	Europe	5	1275 (17.2%)
	Asia	2	471 (6.4%)
	Latin America	2	450 (6.1%)
Center	Single	6	977 (13.2%)
	Multi	17	6429 (86.8%)
Aneurysm			
Size (mean, mm) 8.3 ± 4.5		23	7406
Mean dome < 10 mm		15	4440 (60.0%)
Mean dome > 10 mm		8	2966 (40.0%)
Mean neck < 4 mm		2	564 (7.6%)
Mean neck > 4 mm		9	3015 (40.7%)
Unknown neck length		12	3827 (51.7%)
Location			
Anterior circulation		21	5489 (74.1%)
ICA		4	2169 (29.3%)
Ophthalmic		14	1904 (25.7%)
Cavernous		16	699 (9.4%)
Clinoid		8	466 (6.3%)
MCA		9	191 (2.6%)
ACA/ACoA		4	60 (0.8%)
Posterior circulation		12	461 (6.2%)
Vertebral		5	142 (1.9%)
Basilar		3	133 (1.8%)
PCA/PCoA		7	186 (2.5%)
Unknown/other location		—	1456 (19.7%)
Morphology		15	5085 (68.7%)
Saccular		15	4129 (55.8%)
Fusiform		15	754 (10.2%)
Dissection		8	160 (2.2%)
Blister		5	42 (0.6%)
Unknown morphology		8	2321 (31.3%)

Abbreviations: ACA: anterior cerebral artery; ACoA: anterior communicating artery; ICA: internal carotid artery; MCA: middle cerebral artery; PCA: posterior cerebral artery; PCoA: posterior communicating artery.

**Table 3:** Occlusion data from angiographic follow-up.

		*N* of CO	*N* of followed	Rate, 95% CI	*I* ^2^	*p* value of *I*^2^
Total^a^		4491	5715	0.78 (0.75, 0.82)	90.6%	<0.001^∗^
Angiographic follow-up						
AFU < 6 months		520	763	0.68 (0.65, 0.72)	0.0%	0.576
AFU at 6 months		2996	3828	0.78 (0.77, 0.80)	9.7%	0.341
AFU 6~12 months		1389	1554	0.90 (0.88, 0.92)	34.4%	0.133
Subgroup analysis of AFU at 6 months						
FD	PED	2486	3187	0.78 (0.77, 0.80)	0.0%	0.921
	Other FD	510	641	0.79 (0.72, 0.86)	78.4%	0.010^∗^
Age (year)	>55	442	572	0.78 (0.77, 0.80)	28.3%	0.160
	<55	2554	3256	0.77 (0.74, 0.81)	0.0%	0.937
Dome (mm)	<10	2016	2567	0.79 (0.77, 0.80)	0.0%	0.971
	>10	508	677	0.75 (0.72, 0.78)	0.0%	0.779
Center	Single	160	210	0.76 (0.70, 0.82)	—	—
	Multi	2836	3618	0.79 (0.77, 0.80)	12.0%	0.316

^a^Total outcome of studies with the maximum AFU number. ^∗^Significant heterogeneity. Abbreviations: AFU: angiographic follow-up; CO: complete occlusion; FD: flow diversion; PED: pipeline embolization device; 95% CI: 95% confidence interval.

## Data Availability

The relevant data are available through the corresponding author upon reasonable request.

## Abbreviations

ACA: Anterior cerebral artery
ACoA: Anterior communicating artery
ICA: Internal carotid artery
MCA: Middle cerebral artery
PCA: Posterior cerebral artery
PCoA: Posterior communicating artery
AFU: Angiographic follow-up
CO: Complete occlusion
FD: Flow diversion
PED: Pipeline embolization device
95% CI: 95% confidence interval
mRS: Modified Rankin Scale
HEs: Hemorrhagic events
IEs: Ischemic events

## Supplementary Materials

Supplementary Materials Supplement 1: STROBE scale assessment of all included studies. Supplement 2: basic characteristic of included studies.
