# Supplementary material for: Whether Intracranial Aneurysm Could Be Well Treated by Flow Diversion: A Comprehensive Meta-Analysis of Large-Sample Studies including Anterior and Posterior Circulation
Source: Biomed Res Int. 2021 Mar 8;2021:6637780. doi: 10.1155/2021/6637780 (PMC7969082; doi:10.1155/2021/6637780)
Supplement: Supplementary Materials — Supplement 1: STROBE scale assessment of all included studies. Supplement 2: basic characteristic of included studies. [file 6637780.f1.doc]

**Supplement 1: STROBE scale assessment of all included studies**

| **Author** | **Year** | **STROBE scale /22** |
| --- | --- | --- |
| **Torres et al.** | 2018 | 16/22 |
| **Silva et al.** | 2018 | 16/22 |
| **Saatic et al.** | 2012 | 15/22 |
| **Potts et al.** | 2017 | 15/22 |
| **Petr et al.** | 2016 | 15/22 |
| **Briganti et al.** | 2012 | 16/22 |
| **Becske et al.** | 2013 | 15/22 |
| **Kallmes et al.** | 2017 | 16/22 |
| **Brinjikji et al.** | 2017 | 16/22 |
| **Pumar et al.** | 2017 | 15/22 |
| **Wakhloo et al.** | 2015 | 15/22 |
| **Kim et al.** | 2014 | 15/22 |
| **Delgado et al.** | 2017 | 16/22 |
| **Sweid et al.** | 2018 | 16/22 |
| **Yakovlev et al.** | 2015 | 15/22 |
| **Brasiliense et al.** | 2018 | 16/22 |
| **Trivelato et al.** | 2018 | 15/22 |
| **Piano et al.** | 2013 | 14/22 |
| **Killer et al.** | 2018 | 16/22 |
| **Gupta et al.** | 2018 | 17/22 |
| **Dmytriw et al.** | 2019 | 16/22 |
| **Griessenauer et al.** | 2018 | 16/22 |
| **Adeeb et al.** | 2017 | 16/22 |

Supplement 2: basic characteristic

| **Year** | **Author** | **country** | **Device** | **Age** | **Blind** | **Center** |
| --- | --- | --- | --- | --- | --- | --- |
| **2018** | **Torres et al.** | Argentina | SFD | 51 | NA | multi |
| **2018** | **Silva et al.** | USA | PED | 55.3 | Yes | single |
| **2012** | **Saatic et al.** | Turkey | PED | 49.2 | NA | Single |
| **2017** | **Potts et al.** | USA | PED | 55.6 | NA | single |
| **2016** | **Petr et al.** | Austria | FD | 55.7 | NA | single |
| **2012** | **Briganti et al.** | Italy | SFD or PED | 60 | NA | multi |
| **2013** | **Becske et al.** | USA | PED | 57 | NA | multi |
| **2017** | **Kallmes et al.** | USA | PED | 57.4 | NA | multi |
| **2017** | **Brinjikji et al.** | USA | PED | 55.5 | NA | multi |
| **2017** | **Pumar et al.** | Spain | SFD | 56.2 | NA | multi |
| **2015** | **Wakhloo et al.** | USA | Surpass FD | 57.1 | NA | multi |
| **2014** | **Kim et al.** | USA | PED | 53.2 | NA | multi |
| **2017** | **Delgado et al.** | USA | PED | 55.4 | NA | multi |
| **2018** | **Sweid et al.** | USA | PED | 56.3 | NA | multi |
| **2015** | **Yakovlev et al.** | Russia | Stents FD | 51 | NA | single |
| **2018** | **Brasiliense et al.** | USA | PED | 55.7 | NA | multi |
| **2018** | **Trivelato et al.** | Brazil | PED | 51.89 | NA | multi |
| **2013** | **Piano et al.** | Italy | PED and SFD | 53 | NA | single |
| **2018** | **Killer et al.** | Austria | PED | 54 | NA | multi |
| **2018** | **Gupta et al.** | USA | PED | 57.8 | Yes | multi |
| **2019** | **Dmytriw et al.** | USA | PED | 57 | NA | multi |
| **2018** | **Griessenauer et al.** | USA | PED and FRED | 55.2 | NA | multi |
| **2017** | **Adeeb et al.** | USA | PED | 58 | NA | multi |
